# Reflections on the 7th international Jerusalem conference on health policy in the wake of the Covid-19 outbreak

**DOI:** 10.1186/s13584-020-00436-8

**Published:** 2020-12-28

**Authors:** Shira H. Fischer

**Affiliations:** RAND Corporation, 20 Park Plaza, Suite 920, Boston, MA 02116 USA

**Keywords:** Telemedicine, Health information technology, Innovation

## Abstract

In 2019, a conference in Israel showcased new frontiers in technology in healthcare, highlighting research conducted in Israel as well as across the globe. At the time, no one realized how critical—and ubiquitous—some of these technologies would become. In the wake of a global pandemic, the ability to provide healthcare remotely has become ever more important. We explore some Israeli innovations and consider how healthcare may be permanently changed.

## Background

In September, 2019, when the Israel National Institute for Health Policy hosted The 7th International Jerusalem Conference on Health Policy, very few participants were thinking of an impending pandemic. The theme, “Health and Healthcare in the Age of Innovation,” brought together seven hundred physicians, researchers, and policy-makers to explore the current state of innovation and new technology in healthcare, and imagining a future where in-person was always still possible, if not always convenient.

The COVID-19 pandemic has led to significant changes, at least temporarily, in the delivery of healthcare. It is now relevant to consider how technology development before the pandemic may be relevant for healthcare during the pandemic and how these technologies may affect the future of healthcare.

## Promising projects presented at the conference

The conference had four tracks: Track A: Data-driven care—realizing the promise; Track B: “Uber’ization” of healthcare—dream or nightmare?; Track C: Innovation in health economics, and the economics of healthcare innovation; and Track D: New challenges and threats in the age of innovation. We bring here a few examples of research building on Israel’s strengths, particularly around data and innovation, from the 2019 conference in Jerusalem [1].

Israeli researchers can apply big data techniques in part because they have access to big data [2]. In one project based at Maccabi Health, a health fund which covers about a quarter of Israelis [3], researchers used machine learning to explore the potential role of drugs in patients with hypertension and type 2 diabetes [4]. Benefitting from the comprehensiveness of the data available to them about diagnoses and medications, they explored the impact of existing medications without requiring any new studies or exposures. Promising new treatments were identified, such as statins and proton pump inhibitors for blood pressure. This promising approach to identifying new treatments puts no patients at risk, but relies on large and reliable datasets.

Enabling these kind of efforts is not only the quarter-century of medical records available to Maccabi, but also the addition of molecular and genetic data. Researchers reported on the first year of the first population-basedbiobank in Israel, which opened in 2017 and has over 100,000 vials stored [5]. This rich and growing dataset will enable further research like the machine learning described above, with the addition of potential genetic insights leading to personalized medicine recommendations.

In other work, surveys were used to gather information from patients and providers about telehealth. For example, in one study, researchers conducted a survey using an approach called a “discrete choice” experiment, which enables teasing out the factors that may influence as decision. By asking patients specific questions about video versus in-person clinic visit options, researchers were able to discern that patients cared about time to next available appointment; time in-line before consultation; relationship to primary care physician (PCP); and quality of consultation as they decided between in-person and remote [6]. For PCPs, time in-line before consultation; patient’s self-management ability; consultation purpose; and quality of consultation were all factors to be considered in order to increase uptake of videoconferencing.

Qualitative data is also important to informing the design of future interventions. When we think about conducting telemedicine visits, we know that surgery is different than diagnosis or talk therapy and working with the elderly or children poses additional challenges, but we don’t yet know how best to implement these services, when they’re most effective, and how to improve the experience for patients and providers. In work done at the Clalit health fund, which insures approximately half of Israelis, researchers analyzed the physician experience in a pediatric telemedicine triage service [7]. They identified the challenges physicians face with using telemedicine, such as treating unfamiliar patients and technological challenges, while finding that the physicians had positive experiences and felt they could successfully triage patients remotely.

It’s not just medical appointments that we might want to have at a distance. Psychotherapy, patient education, and even physical therapy may be possible remotely. An increasing number of applications are designed to enable treatment with a person or directly through the app, but there is still more promise for many of these apps than there is robust evidence. A study of a novel mobile application for cardiac rehabilitation is an example of the kind of tools available that may have increasing demand [8]. The app showed effectiveness on exercise capacity, determined by exercise stress test, though there was no comparison group getting traditional cardiac PT.

The conference also highlighted similar work in other countries, offering, for example, a platform to share successes of a telehealth platform in Honduras; artificial intelligence applied to a family health survey in India; and digital diaries to research pathways of hypertension care in the Philippines.

## Technology during a pandemic—and after

We all knew that technology would be increasingly important in healthcare, but few expected the future to come so fast. In the face of a global pandemic that is transmitted via aerosols, the medical system—in Israel as well as in most other countries—has both been overwhelmed by the number of sick patients and at the same time become a risky place for caregivers and patients alike. In response to this pandemic, innovation has accelerated, particularly around tools that allow treatment or monitoring from the home and contact-free interactions. In a report on innovation in response to Covid-19 reflecting the number of innovations in each country’s ecosystem, Israel ranked second, following only the United States [9].

In crisis mode, on the one hand, all resources are focused on addressing the onslaught caused by the novel virus, such that few resources are available for testing and implementing new technology. At the same time, even during the highest peaks, innovation was occurring intrinsically, with providers and other staff inventing out of need, from addressing shortages (leading to PPE reuse and sanitation) to methods of distancing (using markers on windows to avoid entering rooms more than necessary) to technical innovation (ventilator sharing, telemedicine within the hospital, intubation boxes, and more). As evidence gathered for or against certain interventions (for proning [10], for example, and against intubation boxes [11]), practice shifted in light of this. Adoption of technology was also influenced by changes in payment and regulation: in the US, temporary regulatory changes around privacy and payment enabled a great increase in the use of telehealth [12, 13], though the numbers have more recently dropped off [14], and a segment of the population was never able to access those tools even when needed.

At this point in the pandemic, we have an opportunity and a challenge: to rapidly implement technology to maintain access to care in an environment where demand has increased, while limited by the existing infrastructure and knowledge gaps, and to constantly measure the impact to better inform care for the next patients. We must use what we know, such as the research findings described above, to inform our practice moving forward—for example, drug trials for Covid-19 could be derived from the use of big data, like the methods employed at Maccabi, and we can be reassured that children can safely be triaged remotely, based on the experience Clalit’s Pediatrician Online service—as we continue to study cost, satisfaction, and outcomes. As each wave of the pandemic arrives, we should be more prepared, using what we have learned in the past and what we are learning along the way.

But the bigger questions remain for after the pandemic. While it has been shown in the past that telehealth for mental healthcare is effective [15, 16], until the pandemic it was not widely used; we need to study when the default for talk therapy should be via telehealth, perhaps recommending it a priori for established patients or patients who are located at a significant distance from an appropriate therapist. We may find that we continue to triage patients with potentially infectious diseases remotely in emergency rooms to protect healthcare workers. We might move more prenatal and postpartum care to the home, where weight and blood pressure screening can be conducted remotely. We need to determine when in-person care is critical, when virtual is equivalent, and how they may be mixed; for whom these technologies work and for whom they don’t; and when are they equivalent—or potentially better—than our current systems. These are the next questions that researchers must address, to support healthcare beyond this pandemic, into the future.

Israel is well-positioned to test healthcare innovation. Its small size and universal healthcare coverage by one of four HMOs makes it easier to roll-out interventions to a large group of the population in a universal or targeted/randomized way. Also, most electronic records in Israel are digital, with most patients having a full medical history, dating back decades, housed at a single institution. This enables higher quality and continuity of care as well as a rich landscape for research on medical data; though not complete (each fund has its own system, and some hospitals are independent and others are connected to a specific health plan), this generally universal data provides for data analysis, predictive modeling, and subgroup analyses that are difficult to conduct in the United States, where complete data is limited to claims data for those over 65 or in a specific insurance provider, introducing severe limitations and biases, even at the state or municipality level, and available health system data is mostly limited to care sought at that institution. Notable exceptions include the rising number of state-based claims databases (APCDs) and efforts like PCORNET that allow secure pooling of multiple institutional datasets through innovative data merging efforts, and of course the US has a few integrated healthcare systems, such as the Kaiser and the VA, but these approaches also have data limitations, and the integrated systems are not available to most Americans.

Israel is positioned well for a few more reasons. The less clear privacy and ownership policies around use of data for research may make it easier to access health data than in some other countries (though new regulations have been drafted and then held up by the pandemic) [17, 18] and significant government support—through loans rather than equity and for late-stage investment as well as earlier-stage—as well as an active investment community make it a fertile ground for startups. And unlike other countries with universal healthcare, Israel is small and each HMO can operate independently, thus with an even smaller population; whereas the NHS in the UK, insures everyone, is also responsible for more than 66 million people, is one of the top 10 employers in the world [19], and is a huge bureaucracy that has had difficulty moving agilely. There are definitely challenges of the Israeli medical system—including the relatively low doctor to population ratio, high hospital occupancy rates, disparities in care, and a high share of private sources in healthcare financing [20]—but these do not seem to impede innovation.

Indeed, Israel has been called the “Start-Up Nation” [17]. Two months ago, in the midst of the pandemic, Sheba Hospital, the largest hospital in Israel, ran a contest to bring telemedicine to the field of gynecology, offering to partner with and test novel devices and software. The head of the innovation arm noted that the pandemic has sped up adoption of new technologies for remote treatment “and transformed many hospital outpatient services into virtual services.” [21] If the pandemic motivates us to work more quickly to learn to use technology to increase access to and quality of care in ways that can continue into the future, then maybe it will have brought something good.

## Data Availability

Not applicable.
